# Designing an Inclusive Onboarding Program for Academic Ambulatory Care Faculty

**DOI:** 10.7759/cureus.85356

**Published:** 2025-06-04

**Authors:** Arielle Bilek, Amir Rabbani, Joash Wampande, Janet Pregler

**Affiliations:** 1 Department of Internal Medicine, University of California, Los Angeles, Los Angeles, USA; 2 Department of Cardiology, David Geffen School of Medicine, University of California, Los Angeles, Los Angeles, USA; 3 Department of Medicine, University of California, Los Angeles, Los Angeles, USA; 4 Department of Internal Medicine, David Geffen School of Medicine, University of California, Los Angeles, Los Angeles, USA

**Keywords:** burnout, inclusion, job training, medical education, new faculty, onboarding, physician orientation, physician retention, physician wellness, practice efficiency

## Abstract

Academic medical centers are unique healthcare institutions that have a threefold mission of patient care, education, and research. Successful onboarding of new physicians to these institutions requires not only training specific to their roles as clinicians but also additional guidance to navigate the complexities of an academic medical practice as well as teaching and scholarly activities. As more attention is being paid to physician wellness, the onboarding of new physicians should also aim to instill a sense of community and camaraderie, provide practical guidance to ensure the early adoption of efficient clinical practices, and mentor them through the challenges of building a practice. Furthermore, the early establishment of a culture of community engagement and inclusive excellence fosters a sense of belonging that can result in enhanced collaboration, creativity, and, ultimately, organizational success. We present a practical guide for creating a physician onboarding program modeled for an internal medicine department within an academic medical center with broad geographic practice locations, including primary and specialty care.

## Introduction

Onboarding programs play a crucial role in the successful integration of new physicians into academic medical organizations, helping new faculty become familiar with their roles, responsibilities, and organizational culture during their first year [1,2]. Traditional onboarding programs often consist of a brief orientation, such as online modules, a series of introductory lectures, or short-term shadowing experiences, delivered over a few days or weeks. While these models provide a foundational overview, they may not offer the sustained support needed to help new faculty navigate the complexities of academic medical practice over time. Enhanced support, training, and guidance are crucial for newly appointed physicians, especially during their initial year at a new institution or in a new attending role [3]. Longitudinal onboarding programs can be used as a preventative measure to mitigate physician burnout and to establish and reinforce a culture of community engagement, and inclusive excellence, providing a forum for institutions to move from the triple aim (clinical care, research, and teaching) to the quadruple aim, with the added goal of improving the experience of the teams that deliver care [4-9]. Although the many advantages of onboarding programs have been recognized, there is a paucity of peer-reviewed, published, comprehensive guides for developing a robust physician onboarding program tailored to academic medical centers with diverse missions. We present an inclusive, year-long onboarding program designed for new faculty members entering internal medicine practice within a large, geographically diverse academic health system, with a primary emphasis on ambulatory care. The principles outlined here can be easily customized to suit other medical specialties, hospital-based physicians, advanced practice providers, and nonacademic institutions.

## Technical report

Initiating an effective onboarding program

Establishing Mission and Leadership Support

**Figure 1:**
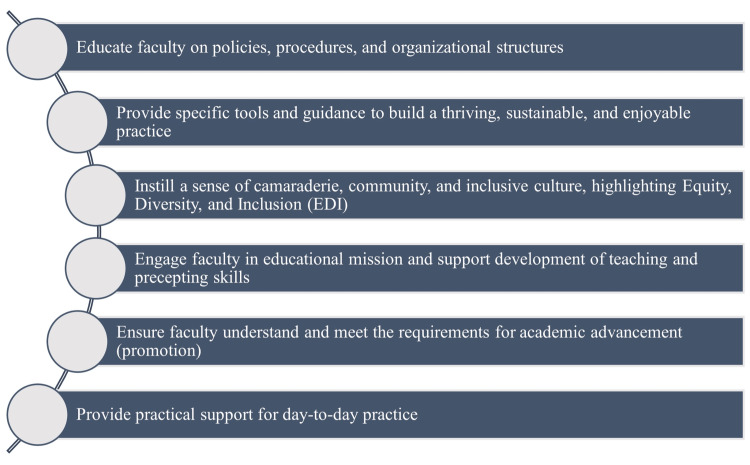
Steps for implementing a physician onboarding program Essential components of an effective onboarding process include educating faculty on policies and structures, providing tools for a sustainable practice, fostering a sense of community with an emphasis on community engagement, involving faculty in educational missions, ensuring clarity on academic advancement requirements, and offering practical day-to-day support Image credit: This is an original image created by the author Joash Wampande

The initial step in developing an effective physician onboarding program involves identifying the onboarding program's mission and securing leadership buy-in and investment. An effective onboarding program should be made mandatory, which necessitates financial support to compensate for the opportunity cost of patient hours lost during participation by the faculty. Onboarding should be grounded in the organizations' mission statement and strategic plan, as well as the department's objectives for physician productivity, professionalism, and retention (Figure 1).

We recommend identifying physician leads with protected time to spearhead the program, aiming for diversity in academic rank, clinical specialty, and geographical practice location within the department. Our team comprises a junior faculty primary care physician at the main campus, a mid-career cardiologist in a community setting, and a senior primary care physician who is a member of the clinical leadership team in our department. For the purposes of our program, junior faculty are defined as those within the first eight years of independent clinical practice, typically holding the titles of Clinical Lecturer or Assistant Clinical Professor. Mid-career faculty generally have 8-16 years of experience and often serve as Associate Clinical Professors. Senior faculty have over 16 years of experience and frequently hold Full Clinical Professor titles, often serving in departmental or institutional leadership roles. A strong and well-sourced administrative team is essential, as large amounts of administrative time are necessary to ensure clinical schedules are coordinated with educational activities, maximizing faculty participation. As our departmental leadership has recognized the value of effective onboarding, our administrative team has steadily grown to include a full-time program manager, as well as program coordinators who manage the program administrative operations.

Onboarding program overview

Program Structuring and Scheduling

Our onboarding program includes approximately 35 hours of general didactic and small group activity contact time, distributed over 11 sessions (general sessions), with about 25 additional hours in specialty-specific small groups, coaching, and other activities as described below. We chose a year-long format to allow onboarding to occur in manageable segments that align with the evolving needs of new physicians throughout their first year. Our general onboarding meetings occur monthly, starting from July at the beginning of the academic year and continuing through June of the following calendar year, encompassing a full academic year. Previously, all sessions were conducted in person before the COVID-19 pandemic; however, the current format involves a blend of video remote and in-person sessions. Each session is scheduled within a designated four-hour block, typically from 8 am to 12 pm. Clinic managers are responsible for blocking this time on the schedules to ensure no patients are scheduled during these sessions.

Participation and Logistics

Our onboarding program caters to an approximate annual cohort of 100 physicians. Our program accepts onboarding physicians (referred to as "onboarders") on an ongoing basis from July to June during each academic year. Attendance at all onboarding sessions is mandatory following the onboarder's official start date, except in cases where they are ill, attending to inpatient service, excused, or other clinical care responsibilities that cannot be reasonably rescheduled. For onboarders commencing their engagement later in the academic year, it is encouraged, but not mandatory, for them to participate in sessions before their official start date. If onboarders have missed more than six months of material, it becomes obligatory for them to attend the missed sessions during the subsequent academic year. If they have missed less than six months of material, they have the option to choose whether to attend the missed sessions and/or review the online recordings and materials.

In-person and video sessions are recorded when logistically feasible. To prevent scheduling conflicts, all session dates are established before the start of the academic year. While we do not currently offer duplicate live sessions, recordings and session materials are made available to accommodate those who cannot attend due to clinical obligations or part-time schedules. This proactive approach helps accommodate faculty members working in various locations and prevents unintended conflicts with clinical care activities.

General sessions typically include 80-100 onboarding physicians per year, with two to four administrative coordinators present per session. Instructional sessions are primarily led by junior and mid-career faculty, with oversight from senior faculty and departmental leadership.

Program organizational structure

**Figure 2:**
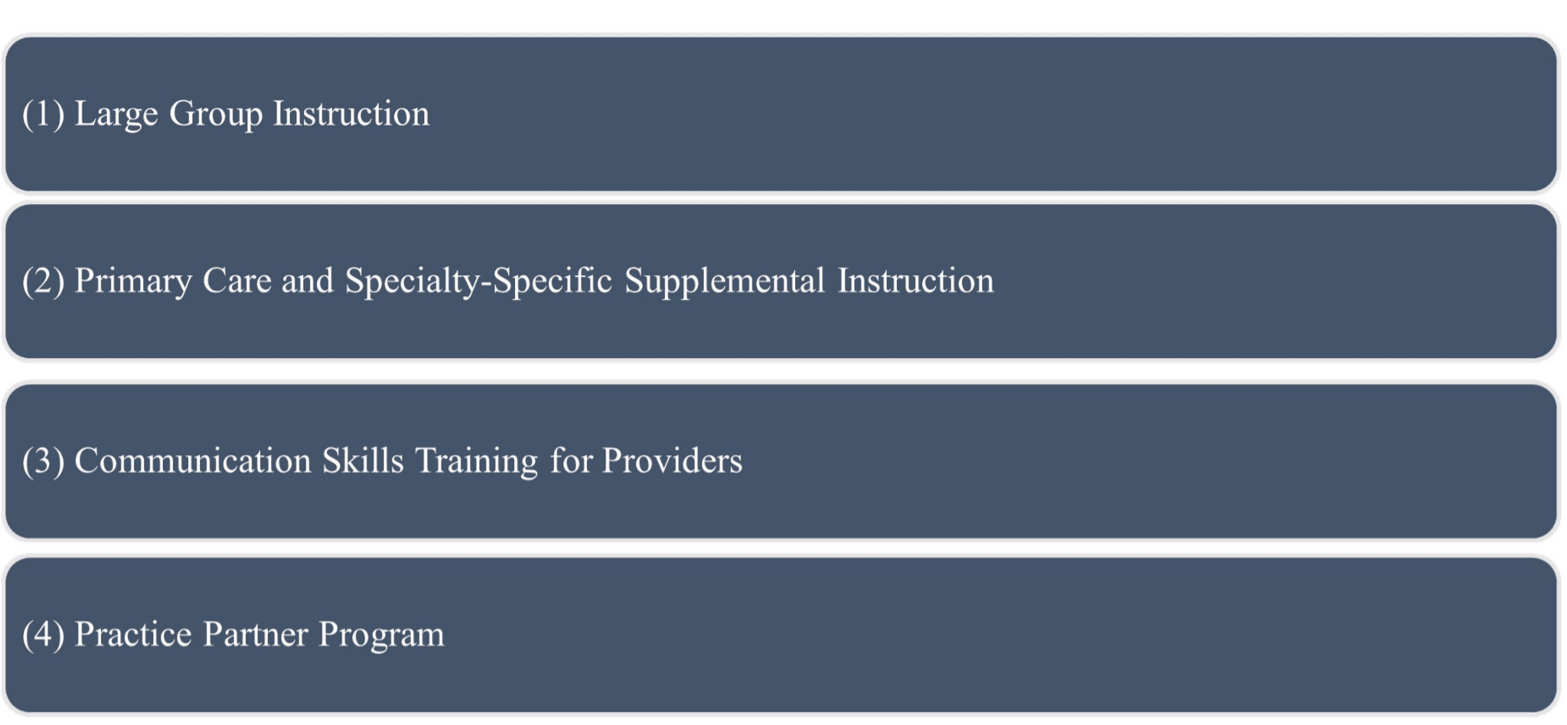
Components of the physician onboarding program The four key components of the onboarding process are a large group instruction for broad foundational training, primary care and specialty-specific supplemental instruction for tailored learning, communication skills training for providers to enhance patient interaction, and a practice partner program for ongoing support and collaboration Image credit: This is an original image created by the author Joash Wampande

Our comprehensive onboarding program consists of four primary components, including large group sessions covering essential content, primary care, and specialty-specific training tailored to these needs, communication skills development workshops, and informal near-peer mentorship, “practice partner program (Figure 2).

Large Group Instruction

**Figure 3:**
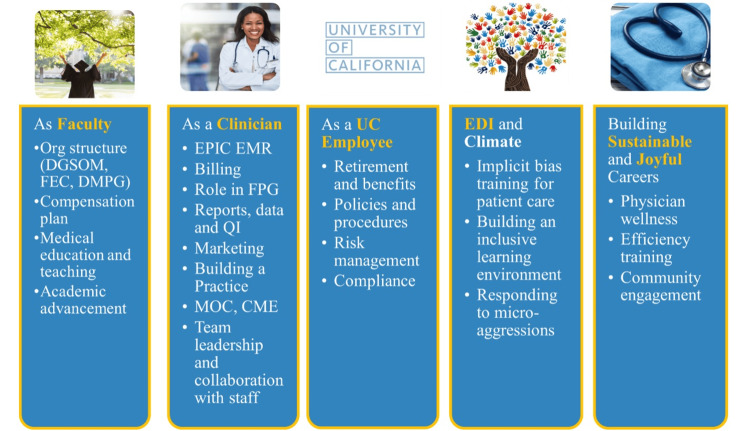
Focus areas for physician onboarding at UCLA Multifaceted roles and responsibilities covered in the onboarding program, including faculty roles, clinical skills, UC employee policies, inclusive excellence and climate initiatives, and strategies for building sustainable careers DGSOM: David Geffen School of Medicine; FEC: Faculty Executive Committee; DMPG: Department of Medicine Practice Group; EMR: electronic medical record; FPG: faculty practice group; MOC: maintenance of certification; EDI: equity, diversity, and inclusion; CME: continuing medical education; UCLA: University of California, Los Angeles; UC: University of California Image credit: This is an original image created by the author Joash Wampande

The content for large group instruction can be categorized into five main areas: 1) training for the role of faculty member, 2) training for the role of clinician, 3) employee information, 4) establishing community engagement and inclusive excellence, and 5) fostering a sustainable and rewarding career (Figure 3). The selection of content primarily focuses on familiarizing faculty with the four pillars while allowing for adaptations to suit the specific requirements and context of each clinical division.

Stakeholder Involvement and Inclusive Excellence

The onboarding program fosters collaboration throughout the health system, engaging key stakeholders such as departmental leadership (including clinical chiefs of subspecialty divisions, regional ambulatory medical directors, and the department chair and vice-chairs), medical school leadership, graduate medical education leadership, equity, diversity, and inclusion (EDI) leadership, physician informaticists, administrative leadership, marketing, finance, legal, human resources/academic personnel, and other subject matter experts. Inclusive excellence topics are a theme throughout all sessions, rather than being presented as “stand-alone” experiences.

Logistics, Community Building, and Session Features

We utilize a large conference center on campus for all our in-person onboarding sessions. The venue offers dedicated areas for booths dedicated to providing one-on-one information on retirement benefits, marketing, and information technology, as well as professional headshot sessions for faculty members' online profiles.

We strive to promote a sense of community among attendees and to minimize barriers to participation. Before each session, onboarders receive a comprehensive reminder email, including detailed parking instructions and a conference center map. Meals are provided to participants. Lactation rooms are provided, and overnight lodging is offered for onboarders who commute more than 40 miles from the venue. To facilitate networking, name badges with location and specialty are required at all sessions.

Sessions feature various elements to enhance engagement, such as breakout sessions designed to encourage discussion, polling, laminated billing crib cards, policies and procedure documents, important articles, and individualized information to assist in marketing their practices.

Primary care and specialty-specific supplemental instruction

**Figure 4:**
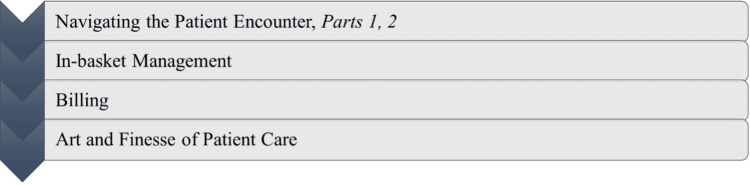
Topics in the supplemental onboarding sessions Core topics addressed in the supplemental sessions for physicians: navigating the patient encounter (Parts 1 and 2), in-basket management, billing, and the art and finesse of patient care, designed to provide practical, role-specific guidance for new physicians Image credit: This is an original image created by the author Joash Wampande

Primary care and specialty-specific supplemental instruction originated from the understanding that the daily tasks and responsibilities differ depending on subspecialty. Primary care and specialty-specific supplemental instruction aims to deliver new physicians timely, practical, and role-specific guidance. Its purpose is to enhance the quality of patient care, optimize comprehension and utilization of the healthcare system, establish effective clinical practices at an early stage, and foster a sense of community and support. We refer to this as “Supplemental Instruction” or “Supplemental Onboarding Program” at our institution (Figure 4). To ensure materials were delivered by knowledgeable instructors, we enlisted individuals known as “Supplemental Instructors” or “Division Champions” from every clinical division in the department. The selection process has evolved to what is now an application process with input from the clinical leadership of each division.

To ensure consistent training across divisions, we adopted a train-the-trainer methodology, wherein standardized guides are created for each session and supplemental instructors are trained by the onboarding physician leadership on how to tailor the curriculum to their specialty. These training sessions are typically conducted during lunchtime, spread across one to two separate days to accommodate the schedules of all the supplemental instructors. They are also recorded for later reference.

While general onboarding sessions are held in person to promote community-building and interspecialty networking, supplemental sessions are delivered virtually to support schedule flexibility and better accommodate specialty-specific content. These remote sessions are scheduled by the instructor, though we encourage a 1:00-2:00 pm time slot, as both instructors and participants often face delays following morning clinic sessions. During the session, instructors follow a standardized guide and use screen sharing to demonstrate electronic medical record (EMR) workflows when relevant. To protect patient privacy, all instructors receive training on how to access and use the EMR training environment appropriately.

During the academic year, a series of five supplemental sessions are conducted, focusing on the following topics: mastering the patient encounter part (two sessions), efficient in-basket management, effective billing practices, and the art of patient care. Moreover, every instructor is granted the authority to adapt the curriculum to their respective subspecialty, allowing them to incorporate insights gained from their day-to-day practice.

Communication skills training

Our university faculty practice group actively supports communication skills training, which is provided to all new physicians throughout our health system. The training consists of a comprehensive two-part workshop on patient communication. The first workshop focuses on goal and agenda setting, while the second addresses the effective handling of challenging patient encounters. The workshops are expertly led and developed by an outside consultant.

Practice partner program

The Practice Partner Program connects onboarders with junior faculty members who can provide guidance and support for day-to-day clinical or operational inquiries. This part of our program was initially elective, with onboarding faculty choosing whether or not to participate. However, based on feedback from onboarding faculty, this is now mandatory. Practice partners are not involved in the evaluation of onboarders and typically do not hold any institutional leadership positions. They are suggested by clinical leaders in their divisions or regions. Each practice partner is assigned no more than two to three onboarders. The practice partner role encompasses various responsibilities, such as providing guidance on available resources for patients. This encompasses counseling centers, treatment centers, and subspecialty urgent care centers. Additionally, the role involves assisting with specific situations, such as advising on the most effective course of action for abnormal results in our system, which may involve referring to specialists or conducting further evaluations. Additionally, the practice partner addresses billing inquiries and handles other minor clinical or logistical questions. The practice partner program fills the void where new faculty may have a question too “small” to ask their leadership, but that cannot easily be found online or through point-of-care resources. The time commitment of a practice partner is generally very small and will vary depending on the needs of that onboarder. There is no formal structure to the program other than requiring a one-time meet-and-greet at the start of the relationship. Practice partners are also required to attend one or two noon-time virtual sessions with peers and the onboarding team to receive a welcome and provide feedback. The onboarder is then encouraged to contact their practice partner as much or as little as is helpful to them.

Soliciting feedback for improvement

After each onboarding session, we provide a QR code (on a poster or PowerPoint slide, Microsoft Corporation, Redmond, WA) for onboarders to complete a brief survey about the session. We then send a follow-up email containing session resources, links to recordings, and the five-minute evaluation survey again if they had not filled it out already. This feedback loop is crucial for continuous improvement, as it allows us to gauge the needs of our onboarders effectively. Furthermore, the evaluations contribute to the professional development and promotion of our presenters. These evaluations are also utilized in presentations to leadership, where we showcase session ratings and gather commentary about the program. Table 1 presents examples of onboarder comments from the 2023-2024 academic year.

**Table 1:** Feedback on the physician onboarding program Summary of participant feedback on the onboarding program from the 2023-2024 academic year, categorized into three themes: overall program experience, networking and collaboration, and the usefulness of specific topics. Comments highlight the program’s role in facilitating transition, providing practical tools, fostering connections, and delivering valuable training, particularly on topics like EPIC EMR and in-basket management EMR: electronic medical record; UCLA: University of California, Los Angeles

Feedback theme	Participant comments
Overall program experience	"Team is honestly great, and I really love the idea of an onboarding program. I thought it was helpful to get me acquainted with UCLA and made my transition far more comfortable." "More Zoom, did not like driving down and then being expected to drive 2 plus hours for the clinic." "Excellent sessions. Thank you for your passion on this. As a new faculty, I did get tremendous help in understanding some of the difficult topics." "Staff and presentations were excellent and felt really cared for in terms of providing us with tools to succeed in our practice." "This was a wonderful year-long program with some very helpful courses, especially learning how to manage an in-basket. I did enjoy meeting people in person but did also find Zoom was very convenient as well. This was an excellent year-long program, and I am happy to have participated! Amazing! Sessions were very welcoming and fun to attend." "Great program and engaged leaders!"
Networking and collaboration	"I think the most helpful part was connecting with other new physicians and learning tips and tricks from them about responding to patient messages, efficiency, etc. The casual small groups were the best, because after we finished the task at hand, we would brainstorm and share tips about practice, and that part was great." "One element that might be helpful is to learn from more individuals on how they structure their day to tackle the clinical volume." "It has been a great program. Great instruction and organization, and allowed in-person interaction, especially coming from a remote clinic."
Specific topic usefulness	"The onboarding sessions were well planned. I especially appreciated the sessions on using EPIC EMR and optimizing in basket. I thought the topics were very useful." "There are some overlapping topics with the large group and supplemental sessions. If this is intended for repetition, then no problem!" "Fantastic programming and a huge help to MDs like me that come from outside the UCLA system." "Most of my knowledge came from residency here, less so onboarding, but it was a nice complement and way to start practice here." "Very helpful, on-the-job practical tips for addressing the common issues in clinical practice."

The program was evaluated using a 6-point Likert scale (5 = extremely well, 0 = I didn’t attend), with results summarized in Table 2. A total of 64 out of approximately 100 onboarders completed the survey, yielding a response rate of 64%. Mean scores were calculated for each session topic, excluding participants who indicated they did not attend. Topics with the highest average ratings included physical wellness: Building a sustainable and joyful career, and EDI and climate, both scoring 4.38 out of 5. Topics such as Compensation (3.25) and Academic Advancement (3.71) received comparatively lower scores, identifying potential areas for future content improvement. Overall, the consistently high mean scores across most categories reflect strong participant satisfaction with the onboarding content.

**Table 2:** Ratings of the physician onboarding program topics (2023-2024 academic year), including mean scores Survey responses were collected using a 6-point Likert scale (5 = extremely well to 0 = I didn’t attend). The table presents the number of responses in each category for each topic, as well as the mean score calculated by excluding “0” responses. A total of 64 out of approximately 100 onboarding physicians responded (64% response rate). Topics are listed in descending order by average rating to highlight areas of strength and opportunities for program enhancement EDI: equity, diversity, and inclusion; EMR: electronic medical record; CME: continuing medical education; MOC: maintenance of certification

Topic	Extremely well (5)	Very well (4)	Moderately well (3)	Slightly well (2)	Not well at all (1)	Didn’t attend (0)	Mean score
Physical wellness	30	31	1	1	1	0	4.38
EDI and climate	25	38	1	0	0	0	4.38
Compliance	23	38	2	0	0	1	4.33
Retirement and benefits	17	34	8	2	0	3	4.08
EPIC EMR	16	33	10	2	2	1	3.94
CME and MOC	15	32	11	3	2	1	3.87
Clinician team leadership	15	40	6	2	1	0	4.03
Medical education and teaching	14	30	14	3	0	4	3.9
3M M*modal	14	28	12	2	2	6	3.86
Clinician marketing	10	29	15	2	2	6	3.74
Organizational structure	8	35	17	3	1	1	3.72
Risk management	6	41	10	1	1	5	3.85
Academic advancement	5	38	20	2	0	0	3.71
Compensation	2	29	22	7	5	0	3.25

## Discussion

This report describes a year-long onboarding program for new physicians at an academic medical center, designed to support ambulatory care faculty through large group instruction, specialty-specific training, communication skills workshops, and a Practice Partner Program. The program is aligned with the Quadruple Aim, with a particular focus on reducing physician burnout and fostering a sense of community. Participant feedback from the 2023-2024 academic year (Tables 1, 2) reflected high satisfaction with key sessions, including EPIC EMR training and in-basket management, which received average ratings of 4.38 on a 5-point Likert scale. These results align with findings from Bodenheimer and Sinsky [8], who emphasize that reducing administrative burden is essential to preventing burnout, affecting nearly half of all physicians, and improving care quality.

High satisfaction with the Physical Wellness, EDI, and Climate sessions (mean scores of 4.38 each) highlights the program’s emphasis on physician well-being and fostering an inclusive environment. These results support the findings of Terregino et al. [9], who emphasize that inclusive cultures reduce burnout and can help avoid the high costs associated with physician turnover, estimated at approximately $500,000 per physician. In alignment with Carrau and Janis [4], who underscore the role of mentorship and community-building in mitigating burnout, our program incorporates both a hybrid delivery format and a structured practice partner program to promote peer connection and support. Meanwhile, lower satisfaction with topics such as compensation (mean score 3.25) and clinician marketing (3.74) reflects areas where faculty perceived less value, pointing to opportunities for content revision and improvement. These results echo Bodenheimer and Sinsky [8], who note that resource gaps in primary care can hinder physician engagement and satisfaction.

The train-the-trainer model ensured consistent instruction, and mentorship enhanced collaboration (Table 1), reinforcing Carrau and Janis' [4] focus on social support. The internal medicine focus may limit generalizability, as noted by Terregino et al. [9]. Long-term outcomes, such as retention and quality metrics, require further study.

## Conclusions

This guide offers a practical framework for developing an effective and inclusive onboarding program tailored to academic medical centers, with a focus on ambulatory internal medicine. By emphasizing structured training, integration of EDI, community building, and strong leadership support, the program has demonstrated positive impacts on faculty well-being, performance, and sense of belonging, key elements for achieving the quadruple aim. Designed as a living guide, it can be adapted to institutional priorities and refined through continuous evaluation. We offer three actionable takeaways for other institutions: 1) implement a year-long, multimodal onboarding structure that accommodates diverse faculty needs, 2) integrate equity, wellness, and mentorship longitudinally rather than as standalone elements, and 3) secure administrative support and leadership engagement to ensure program sustainability and growth. Future research could help clarify the long-term effects on faculty retention and patient care outcomes.
